# Changes in Conversational Pattern as a Clinical Trial Outcome Using the I-CONECT Data

**DOI:** 10.1093/geroni/igaf122.1206

**Published:** 2025-12-31

**Authors:** Liu Chen, Chao-Yi Wu, Hiroko Dodge

**Affiliations:** Massachusetts General Hospital, Harvard Medical School, Boston, Massachusetts, United States; Massachusetts General Hospital, Harvard Medical School, Boston, Massachusetts, United States; Massachusetts General Hospital, Harvard Medical School, Boston, Massachusetts, United States

## Abstract

**Background:**

Linguistic measures derived from spontaneous conversations are behavioral indicators linked to cognitive functions. We investigated a natural language processing measure, semantic noise (SN), to quantify conversational patterns and monitor intervention-induced changes.

**Method:**

We analyzed transcriptions from semi-structured conversations in the I-CONECT project (ClinicalTrials.gov: NCT02871921). Participants engaged in 30-minute semi-structured conversations four times per week via online video chats for six months. Mean SN (MSN) from the second and 24th weeks’ conversations represented participants’ conversational patterns at baseline and month six (M6), respectively. Two-tailed t-tests and paired t-tests are used for statistical analysis.

**Results:**

Among participants who completed baseline and M6 video chat interventions, the MCI group’s MSN was significantly higher than the NC group’s (MCI: 2.09 ± 0.34, NC: 1.87 ± 0.35; p = 0.024) at baseline. After six months of intervention, no significant difference was observed between groups (MCI: 1.81 ± 0.47, NC: 1.95 ± 0.47; p = 0.280). In the MCI group, baseline MSN was significantly higher than M6 MSN (paired t-test: p = 0.009), but no significant change occurred in the NC group (paired t-test: p = 0.426).

**Conclusions:**

We demonstrated a conversational pattern difference between the MCI and NC groups at baseline. After six months of conversational interaction, the MCI group’s MSN decreased, shifting from being significantly higher than that of the NC group at baseline to no significant difference at M6. This suggests that following the intervention, the conversation pattern of the MCI group resembled that of the NC group.

